# Assessment of Orofacial Function After Mandibular Angle Harmonization with Hyaluronic Acid: A Longitudinal Observational Study

**DOI:** 10.3390/dj14040241

**Published:** 2026-04-17

**Authors:** Nicole Barbosa Bettiol, Franciele Aparecida de Carvalho, Selma Siessere, Giovana Dornelas Azevedo Romero, Márcio de Menezes, Catia Cristina Janjacomo Martini, Jardel Francisco Mazzi-Chaves, Laís Valencise Magri, Simone Cecilio Hallak Regalo, Marcelo Palinkas

**Affiliations:** 1 Department of Basic and Oral Biology, Ribeirão Preto School of Dentistry, University of São Paulo, Avenida do Café, No Number, Ribeirão Preto 14040-904, Brazil; nicole.bettiol@usp.br (N.B.B.); francielecarvalho@usp.br (F.A.d.C.); selmas@forp.usp.br (S.S.); garomero@usp.br (G.D.A.R.); catiajanjacomomartini@usp.br (C.C.J.M.); simone@forp.usp.br (S.C.H.R.); 2 National Institute-Technology-Translational Medicine (INCT.TM) and INCT Digital Mental Health, Ribeirão Preto 14040-904, Brazil; 3 School of Health Science, State University of Amazonas, Avenida Carvalho Leal, 1777, Manaus 69065-001, Brazil; 4 Department of Restorative Dentistry, Ribeirão Preto School of Dentistry, University of São Paulo, São Paulo, Avenida do Café, s/n, Ribeirão Preto 14040-904, Brazil; jardel.chaves@usp.br (J.F.M.-C.); laisvm@forp.usp.br (L.V.M.)

**Keywords:** mandible, hyaluronic acid, bite force, orofacial pressure, stomatognathic system

## Abstract

**Background:** The relationship between facial aesthetic procedures and changes in the stomatognathic system has attracted increasing interest, motivating investigations into their functional and structural impacts. This longitudinal observational study analyzed molar bite force and orofacial tissue pressure in adults who underwent hyaluronic acid injections in the mandibular angle. **Methods:** Ten adults (eight women and two men; mean age 34.3 ± 11.2 years) with normal occlusion and no temporomandibular disorders were included. The MD Codes guided injection points of 2 mL of hyaluronic acid in the mandibular angle. Maximum right and left molar bite force was measured using a digital dynamometer, and tongue, lip, and cheek pressures were measured with a Pro-Fono Biofeedback device. Assessments occurred before and at 15, 30, and 60 days. Repeated measures ANOVA with Bonferroni correction was applied (*p* < 0.05), and effect sizes and 95% confidence intervals were calculated. **Results:** No statistically significant differences were observed in maximum molar bite force throughout the follow-up period. Regarding orofacial pressures, a significant main effect of time was observed for tongue pressure (*p* = 0.03); however, the effect size was moderate-to-large, and values showed considerable variability across participants. Lip and cheek pressures remained stable over time. **Conclusions:** Hyaluronic acid injection in the mandibular angle did not show clinically detectable changes in maximum molar bite force, suggesting short-term preservation of masticatory function within the 60-day follow-up period. These findings are limited to short-term observations and specific sample characteristics. The observed variation in tongue pressure may reflect adaptive functional adjustments, although variability across participants was considerable.

## 1. Introduction

The face plays a central role in human communication, reflecting emotions, age, and health, and influencing self-esteem. The factors that define facial attractiveness have been studied for centuries across different fields [1]. Research indicates that symmetry, proportionality, and well-defined contours are key characteristics associated with beauty [2,3].

In this context, increased longevity and the growing emphasis on physical appearance have driven the demand for aesthetic interventions, as features such as facial volume, skeletal pattern, dental morphology, and contour are key determinants of perceived beauty [4]. Among these elements, mandibular shape and the definition of the mandibular angle stand out as important markers of femininity and masculinity [5,6].

With the growing appreciation of the lower third of the face, the demand for non-surgical alternatives to enhance this region has increased, driving the use of hyaluronic acid. This biocompatible biomaterial is widely used as a dermal filler due to its ability to hydrate, stimulate collagen and elastin production, and offer different densities and durabilities depending on the degree of cross-linking [7,8]. Hyaluronic acid has been shown to be effective in remodeling the mandibular contour, restoring volume, and correcting asymmetries, with immediate results [9].

However, for the appropriate planning, execution, and evaluation of aesthetic interventions in the lower third of the face, it is important to understand the stomatognathic system, a functional complex composed of static and dynamic structures such as bones, joints, muscles, teeth, and soft tissues, responsible for essential functions such as mastication, swallowing, speech, and mandibular posture [10]. Structural or volumetric changes in the face may interfere with muscular balance, occlusal dynamics, and mandibular biomechanics, thereby affecting the activity of the masticatory muscles and the overall functional efficiency of the system [11].

In particular, the masseter muscle, which has a direct anatomical relationship with the mandibular angle, plays a key role in bite force generation, and its biomechanical behavior may be sensitive to local volumetric modifications. Additionally, neuromuscular plasticity mechanisms may enable adaptive responses of the stomatognathic system to peripheral structural changes, maintaining functional performance even in the presence of anatomical alterations [12].

Thus, although aesthetic procedures with hyaluronic acid offer well-established benefits for facial contouring [13], it is essential to assess their potential functional effects in order to ensure not only harmonious aesthetic outcomes but also preservation of stomatognathic system function. It is important to emphasize that most available studies in this field focus predominantly on aesthetic outcomes, with limited investigation of functional parameters, particularly objective measures such as bite force and orofacial tissue pressure. Furthermore, controlled or randomized studies in this specific context remain scarce, highlighting the relevance of exploratory observational approaches as an initial step in knowledge development.

In light of the above, the aim of this study was to analyze stomatognathic system function in adults undergoing hyaluronic acid injection in the mandibular angle by assessing maximum molar bite force and orofacial tissue pressure. This study was designed as a longitudinal observational investigation, in which each participant served as their own control over time, allowing the evaluation of functional changes following a standardized clinical intervention. The null hypothesis states that this intervention does not promote changes in stomatognathic system function.

## 2. Materials and Methods

### 2.1. Study Population and Sample Characterization

This longitudinal observational study was approved by the Research Ethics Committee of the School of Dentistry of Ribeirão Preto, University of São Paulo, Brazil (protocol code 79298124.2.0000.5419; date of approval: 25 April 2024), and all participants provided written informed consent.

The study was designed as a within-subject longitudinal investigation, in which each participant served as their own control over time. This approach was chosen to reduce inter-individual variability in stomatognathic function and to allow the evaluation of functional changes following a standardized clinical intervention, rather than to establish causal relationships.

Sample size calculation was performed using G*Power software version 3.1.9.2 (Franz Faul, University of Kiel, Germany), based on a reference study that evaluated improvement in chin retrusion after hyaluronic acid treatment compared with a non-intervention control group, measured 12 weeks after the last application by a blinded evaluator [14]. In that study, means (±standard deviations) for improvement of at least one point were 87 (±81.3) in the treated group and 2 (±6.1) in the control group. Based on these parameters, an effect size of 1.08 and a statistical power of 81% were estimated, indicating a minimum required sample size of 10 participants for the present study.

However, it is important to note that this calculation was based on outcomes different from those evaluated in the present study (bite force and orofacial pressure), due to the absence of prior studies with comparable functional endpoints. Therefore, this sample size estimation should be interpreted with caution, and the study should be considered exploratory in nature. Accordingly, it should not be interpreted as evidence of adequate statistical power for the specific functional outcomes evaluated in this study.

Initially, 30 participants were assessed, and after applying the inclusion and exclusion criteria, 10 were eligible and included in the study, comprising eight women and two men, with a mean age of 34.3 ± 11.2 years and a mean body mass index of 23.94 ± 2.35 kg/m^2^. Assessments of stomatognathic system variables were performed at baseline (pre-procedure) and at 15, 30, and 60 days after hyaluronic acid injection in the mandibular angle.

The sample consisted of adults with complete permanent dentition, normal occlusion, good general health, and a body mass index between 18.5 and 25 kg/m^2^. During the follow-up period, surgical interventions and other facial aesthetic procedures were not permitted. Participants with open skin lesions or ulcers, cognitive impairment, neurological diseases, or decompensated systemic conditions were excluded. Participants using fixed or removable dental prostheses, taking muscle relaxants with potential interference in neuromuscular physiology, diagnosed with temporomandibular disorders, with a previous history of facial filler injections, or with known hypersensitivity to hyaluronic acid were also not included. These strict criteria were intentionally adopted to minimize confounding factors that could independently affect stomatognathic function.

ChatGPT (OpenAI, GPT-5 model) was used solely for text translation and the creation of illustrative images. It was not involved in study design, data analysis, or interpretation. The authors reviewed, edited, and assume full responsibility for the final content.

### 2.2. Assessment of Temporomandibular Disorders

The assessment of temporomandibular disorders was performed based on the Diagnostic Criteria for Temporomandibular Disorders (DC/TMD), including evaluation of Axes I and II [15]. The DC/TMD was applied by a single trained examiner. The examiner underwent prior training in the application of the DC/TMD protocol to ensure standardized data collection.

### 2.3. Hyaluronic Acid Injection Technique in the Mandibular Angle

The standardized facial mapping system MD Codes was used to precisely guide the injection points of hyaluronic acid in the mandibular region. This method is based on a specific set of codes aimed at correcting different facial deficiencies, including reduced definition of the mandibular contour [16]. The JAW code was applied specifically to the mandibular angle region. Each code was accompanied by a numerical sequence that guided the order and the exact injection points of hyaluronic acid, aiming to optimize outcomes. The technique also determined the appropriate depth of injection for each code, the minimum volume of product to be used, and the indicated type of instrument (cannula). A total of 2 mL of hyaluronic acid (Voluma Juvederm; Allergan Inc., Irvine, CA, USA) was injected into each hemiface. This hyaluronic acid–based filler, with a concentration of 20 mg/mL, was the first to receive approval from the U.S. Food and Drug Administration (FDA) for the restoration of age-related volume loss in the midface region. It provides effective and safe aesthetic improvements, with reduced downtime and a low rate of complications [17]. In the mandibular angle region (JAW1), 0.5 mL was injected using a cannula; in the preauricular area (JAW2), the same amount of product was applied; and in the mandibular body (JAW3), 1 mL was injected. The use of these three injection points provided effective mandibular definition through a safe, evidence-based technique.

All procedures were performed following a standardized protocol by a specialist in orofacial harmonization, ensuring consistency in injection technique and volume across participants. However, no imaging methods (e.g., ultrasound or 3D analysis) were used to objectively verify filler distribution or persistence, which limits mechanistic interpretation of the functional findings.

### 2.4. Molar Bite Force Analysis

Maximum bite force (N) at the permanent first molars was assessed using a digital dynamometer (model IDDK; Kratos—Industrial Equipment Ltda.., Cotia, São Paulo, Brazil) specially adapted for intraoral use. During bite force recording, participants were seated with their palms resting on their thighs. To ensure biosafety, the dynamometer was disinfected with alcohol and covered with disposable latex finger cots (Wariper, São Paulo, Brazil). Prior to the measurements, all participants received detailed instructions on the operation of the device, ensuring procedural reliability. Three bite force measurements were obtained on both sides (right and left), with a two-minute interval between each measurement. The highest value recorded among the three measurements was considered for subsequent analysis, as it reflects maximal performance capacity, as commonly adopted in previous studies [18]. All measurements were performed by the same examiner following standardized procedures.

### 2.5. Orofacial Tissue Pressure Analysis

The method used to measure pressure exerted by the orofacial tissues was the Pro-Fono Biofeedback device (PLL Pro-Fono, São Paulo, Brazil), which measures lip, tongue, and cheek pressure [19]. The device consists of a pressure sensor and an electronic board. The pressure sensor is connected to a flexible plastic tube, which is attached to an air bulb device. This system was used to measure the pressure exerted by the lips, tongue, and cheeks by means of compressive movements applied to a pressure transducer. The training module provides a biofeedback feature that allows visualization, on a monitor, of the applied pressure (kPa) as a function of time (s). Prior to the examination, participants were seated in a comfortable chair, maintaining an upright posture, with their feet supported on the floor and their hands positioned on their thighs. Measurements were performed three consecutive times, and the mean value of the three trials was used for analysis in order to represent functional consistency and reduce variability [20].

Lip pressure was assessed with the plastic bulb positioned between the lips, and participants were instructed to apply maximum pressure for three seconds with the teeth in occlusion. Tongue pressure against the hard palate was measured by elevating the tongue and compressing the bulb against the palate, also for three seconds. Cheek pressure (right and left) was assessed with the bulb positioned between the molar teeth and the cheek, in the vestibular region, with maximum pressure applied for three seconds. All measurements were performed by the same examiner following standardized procedures.

### 2.6. Statistical Analysis

After data collection of maximum molar bite force on the right and left sides, as well as measurements of orofacial tissues (lips, tongue, and cheeks), data normality was assessed using the Shapiro–Wilk test, which indicated a normal distribution. Statistical analyses were performed using the Statistical Package for the Social Sciences software, version 20.0 (SPSS Inc., Chicago, IL, USA). Repeated-measures analysis of variance (ANOVA) with Bonferroni correction was used to evaluate changes over time, with a significance level set at 5% (*p* < 0.05). In addition to *p*-values, effect sizes were calculated using partial eta squared (η^2^p) to assess the magnitude of the observed effects. Descriptive results were expressed as mean values accompanied by 95% confidence intervals (95% CI), providing an estimate of precision. The interpretation of results considered not only statistical significance but also effect sizes and confidence intervals, particularly given the small sample size. Furthermore, individual trajectories were visually inspected to assess within-subject variability across time points.

### 2.7. Reliability Analysis

The examiner responsible for all measurements underwent prior training and calibration procedures to ensure standardized data collection. Intra-examiner reliability was assessed using the Dahlberg formula, based on two repeated measurements performed in five individuals, with a seven-day interval between sessions. The method error was low, corresponding to 5.21% for bite force and 4.38% for orofacial tissue pressure, indicating good measurement reproducibility.

## 3. Results

**Figure 1:**
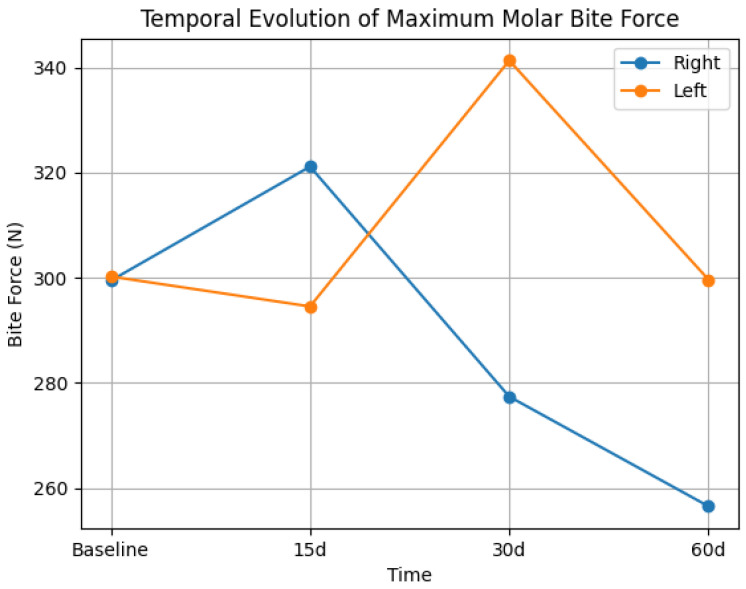
Temporal evolution of maximum molar bite force (right and left sides) at baseline and 15, 30, and 60 days after hyaluronic acid injection in the mandibular angle. No statistically significant differences were observed over time.

Table 1 presents the maximum molar bite force (right and left) at baseline and at 15, 30, and 60 days after hyaluronic acid injection in the mandibular angle. No statistically significant effect of time was observed for maximum molar bite force on either side (right: F = 0.44, *p* = 0.61; left: F = 0.50, *p* = 0.68), with small effect sizes (η^2^p = 0.06 and η^2^p = 0.05, respectively). Mean values remained relatively stable over time for both sides, with no consistent pattern of increase or decrease. Confidence intervals were wide and showed substantial overlap across time points, indicating considerable inter-individual variability. The temporal evolution of maximum molar bite force is illustrated in Figure 1.

**Figure 2:**
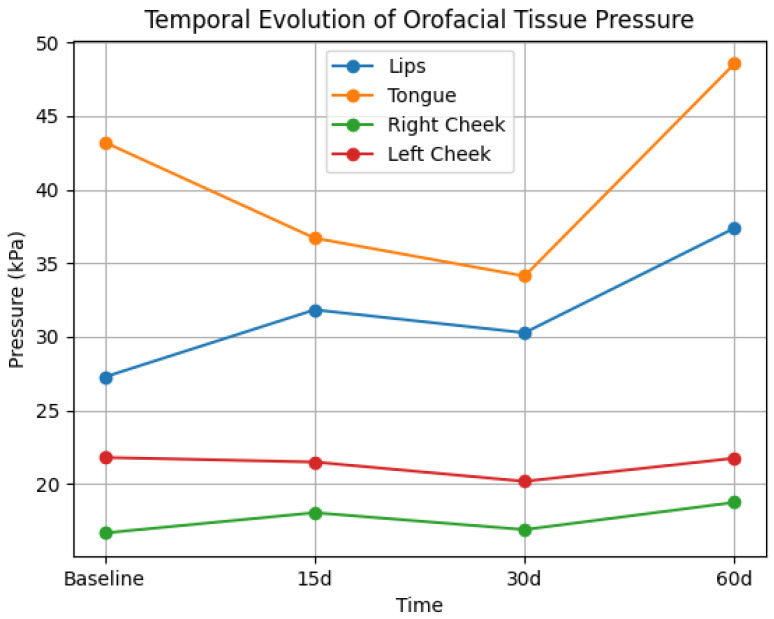
Temporal evolution of orofacial tissue pressure (lips, tongue, and cheeks) at baseline and 15, 30, and 60 days after hyaluronic acid injection.

Table 2 presents tongue, lip, and cheek pressure at baseline and at 15, 30, and 60 days after hyaluronic acid injection in the mandibular angle. No statistically significant effect of time was observed for lip or cheek pressures (*p* > 0.05), with small effect sizes for these variables, indicating minimal influence of the intervention. Mean values remained relatively stable across time points, with no consistent temporal pattern. Tongue pressure showed a statistically significant effect on time (*p* = 0.03), with a moderate-to-large effect size. However, confidence intervals were wide and showed substantial overlap, indicating variability across participants. Overall, wide confidence intervals were observed for all variables, reflecting considerable inter-individual variability. The temporal evolution of orofacial tissue pressure is illustrated in Figure 2.

## 4. Discussion

The null hypothesis of this study was partially rejected, as no significant differences were observed in maximum molar bite force throughout the follow-up period after hyaluronic acid injection in the mandibular angle; however, a statistically significant effect in tongue pressure was observed over time. Given its longitudinal observational design, the findings should be interpreted within an exploratory framework, without establishing causal relationships.

### 4.1. Impact on Bite Force

The results of the present study indicated that hyaluronic acid injection in the mandibular angle did not promote significant changes in maximum molar bite force on either side over the 60-day follow-up period. Within the limits of this study, these findings suggest that the procedure did not produce clinically detectable alterations in maximal force generation during this timeframe. However, given the relatively small sample size, these results should be interpreted with caution, particularly considering the potential risk of type II error, which may have limited the detection of subtle functional changes.

These findings suggest that, under the clinical conditions and filler volume used, the procedure did not significantly interfere with the functional efficiency of the stomatognathic system. However, the absence of statistically significant differences should not be interpreted as definitive evidence of functional invariance. Considering that the masseter muscle has a direct insertion in the mandibular angle region and plays a key role in bite force generation [21], it would be reasonable to expect that local volumetric modifications could influence muscular biomechanics [22].

Recent evidence indicates that the application of hyaluronic acid–based fillers in facial regions may promote changes in tissue displacement patterns. These findings suggest that the introduction of local volume can modify the spatial relationship between muscles and adjacent tissues, thereby influencing force vectors and the biomechanics of muscle contraction [23].

In this context, it would be plausible to assume that volumetric interventions in the mandibular angle region could functionally affect masseter muscle performance within the stomatognathic system. However, the data obtained in this study showed that any structural changes induced by filler injection were insufficient to produce a clinically detectable functional impact on maximum force-generating capacity. It is important to emphasize that this interpretation is limited to the sensitivity of the methods used and the specific conditions of this study.

### 4.2. Comparison with Previous Literature

These findings are consistent with the current understanding that hyaluronic acid–based fillers, when applied in appropriate tissue planes and with controlled volumes, tend to promote predominantly aesthetic modifications, with very limited functional repercussions [17,24,25]. Moreover, the rheological properties of hyaluronic acid, characterized by viscoelastic behavior, allow absorption and redistribution of mechanical loads within the tissue microenvironment [26]. This property may attenuate potential interference with force transmission in the complex arthromyofascial system. Thus, even given the anatomical proximity between the injection site and the insertion of the masseter muscle, the filler material does not appear to compromise the efficiency of masticatory biomechanics. However, these findings should be interpreted cautiously and not extrapolated beyond the evaluated conditions.

In this context, it is noteworthy that the choice of a hyaluronic acid filler with a rheological profile characterized by a high elastic modulus G′, combined with adequate tissue spreadability, was essential to balance structural support and functional preservation. An excessively rigid gel could result in greater local mechanical resistance and potentially interfere with the dynamics of the myofascial planes. In contrast, an overly fluid material would tend to provide lower tissue support and limited aesthetic impact [26]. Thus, the careful selection of the filler may have contributed to explaining the maintenance of bite force observed in this study, while simultaneously enabling aesthetic outcomes compatible with the functional integrity of the stomatognathic system.

### 4.3. Physiological Mechanisms and Adaptation

From a neuromuscular perspective, the masticatory process depends on the integration of central motor commands and sensory afferents. These inputs arise from muscular, fascial, periodontal, and articular mechanoreceptors and are primarily organized by the trigeminal system [27,28]. Peripheral changes in the mandibular region could modify afferent input and require adaptive adjustments in motor control. However, the absence of significant changes in bite force throughout the follow-up period suggests that the stomatognathic system was able to maintain functional performance, possibly through compensatory mechanisms and neuromuscular adaptation, consistent with principles of neural plasticity described for motor systems [29,30]. This adaptive capacity may help explain the maintenance of global functional outputs despite localized structural modifications.

### 4.4. Impact on Orofacial Pressures

In contrast, a statistically significant effect of time on tongue pressure was observed. However, this effect was characterized by a moderate-to-large effect size and considerable variability, as reflected by the wide and overlapping confidence intervals. Therefore, despite being statistically significant, the finding should be interpreted cautiously due to variability across participants. The tongue plays an important role in the balance of the stomatognathic system, actively participating in mastication, swallowing, speech, and maintenance of mandibular posture [19]. Thus, variations in tongue pressure may reflect adaptive processes in response to indirect biomechanical modifications induced by aesthetic intervention in the mandibular region.

The initial reduction observed up to day 30, followed by an increase at 60 days, may be associated with a period of tissue accommodation and progressive neuromuscular reorganization. This pattern may reflect functional adaptation to the new structural conditions. However, this finding should not be interpreted as evidence of a consistent functional change, but rather as a variable response across participants, as it may reflect random variability or a multiple-testing artifact rather than a true physiological adaptation.

The fact that only tongue pressure showed a statistically significant effect over time suggests that this anatomical structure may be more sensitive to changes in intraoral functional space and mandibular dynamics [31]. Unconscious postural adjustments of the tongue may occur in response to subtle modifications in the external mandibular contour. These adjustments may reflect a fine adaptive mechanism of the orofacial system without necessarily producing measurable effects on bite force. Nevertheless, the possibility of type I error cannot be excluded, even with the application of Bonferroni correction.

Experimental evidence indicates that the tongue exhibits high sensitivity to subtle variations in contour and intraoral functional space. It can adjust its posture and the magnitude of the pressure exerted even in response to minimal changes in the oral environment. Such fine adjustments reflect an adaptive mechanism of the orofacial system that may occur in isolation, without necessarily translating into measurable changes in other global functional variables [32].

Mild inflammatory processes and local edema are expected after injectable procedures, even when properly performed. These factors may temporarily interfere with local sensitivity and regional proprioceptive feedback [24]. Such sensory changes may transiently modulate patterns of muscle activation and the functional coordination of orofacial tissues, thereby accounting for initial variations in tongue pressure. As the inflammatory process progressively resolves and the tissues accommodate around the filler, functional reorganization occurs, consistent with neuromuscular adaptation mechanisms described for the masticatory system in response to peripheral perturbations [33].

With regard to lip and cheek pressure, no significant differences were observed throughout the follow-up period, although a trend toward increased mean lip pressure values over time was identified. These findings suggest that the overall functional balance of the perioral tissues was preserved. However, subtle changes may not have been detected due to sample size limitations.

In this context, the functional stability of orofacial structures reinforces the hypothesis that hyaluronic acid injection in the mandibular angle, when performed judiciously, has a low potential for clinically relevant interference with orofacial dynamics [34]. However, the observed variability, particularly for tongue pressure, should be interpreted with caution and not as definitive evidence of functional change.

Consistent with this interpretation, clinical studies indicate that hyaluronic acid filler in the mandibular region, when guided by anatomical knowledge, presents a favorable safety profile, with predominantly mild and transient adverse events and no clinically significant functional repercussions. These findings suggest preservation of the functional stability of perioral and mandibular structures after the procedure [25].

When compared with procedures performed in facial regions with more direct functional involvement, such as the lips, where functional changes have already been reported in the literature [22], the findings of the present study suggest that the mandibular angle represents a region with potentially lower direct functional impact, acting predominantly as a structural area for aesthetic support. This regional difference highlights the importance of analyzing functional effects of aesthetic procedures in a region-specific manner, considering the anatomical and functional particularities of each facial area.

### 4.5. Clinical Implications

From a clinical perspective, the findings of the present study contribute to the understanding of the functional behavior of orofacial harmonization procedures, particularly mandibular angle filler. In this sense, the results suggest that aesthetic interventions in predominantly structural regions may have a low potential for clinically detectable functional impairment under the evaluated conditions, at least in the short term. However, these findings should not be interpreted as definitive evidence of functional safety.

In contrast, the variability observed in tongue pressure suggests that facial aesthetic procedures may induce subtle and potentially transient adaptive responses, which should be understood as part of a morphofunctional accommodation process to facial contour modifications, reinforcing the relevance of an integrated clinical approach that considers both morphological aspects and potential functional repercussions.

### 4.6. Methodological Limitations

The study presents limitations, including the small sample size and the longitudinal observational design, which restrict the generalizability of the findings. The absence of a control group prevents causal inferences and the differentiation between intervention effects and natural variability or learning effects. The reduced sample size also decreases statistical power, increasing the risk of type II error and limiting the detection of subtle functional changes, while not allowing the exclusion of regression to the mean. In addition, the wide confidence intervals and the small effect sizes observed for most variables indicate considerable uncertainty regarding the magnitude of the effects, reinforcing the exploratory nature of the findings. The inclusion criteria (normal occlusion, absence of temporomandibular disorders, and controlled body mass index) limit the applicability of the findings to broader clinical populations. The sample size calculation was based on outcomes different from those evaluated, and no imaging methods were used to verify filler distribution. Additionally, the methodological choices for outcome measurement (maximum bite force values and mean orofacial pressure) reflect different aspects of functional performance. Furthermore, although a statistically significant effect was observed for tongue pressure, this finding was not consistent across participants and should be interpreted considering the observed variability rather than a consistent functional pattern. These limitations also hinder the understanding of mechanistic relationships between filler application and functional outcomes. Despite these limitations, the study has exploratory relevance, serving as a foundation for future investigations. Future studies are recommended to use randomized controlled (or split-face) designs, include appropriate comparator groups, and adopt predefined clinically meaningful thresholds to improve causal inference and the clinical relevance of the findings.

The 60-day follow-up primarily reflects short- to mid-term functional responses after aesthetic procedure and does not capture the full duration of hyaluronic acid persistence or long-term tissue remodeling. Although this period allows observation of early functional adjustments, previous studies suggest that filler effects tend to stabilize early, with minimal changes after approximately 60 days, indicating early stabilization of effects [25]. Nevertheless, longer follow-up periods are necessary to confirm the persistence and long-term functional implications of these findings.

Conceptually, the findings of the present study reinforce that orofacial harmonization should not be understood exclusively as an aesthetic intervention, but rather as a potential morphofunctional modulation of the stomatognathic system. However, these effects were not consistently observed across the evaluated variables and should be interpreted with caution. This perspective highlights the need for clinical protocols that incorporate functional assessment before and after the procedure. It also reinforces the importance of an interdisciplinary approach that integrates aesthetics, function, and systematic functional follow-up.

## 5. Conclusions

Hyaluronic acid injection in the mandibular angle did not show clinically detectable changes in maximum molar bite force over the 60-day follow-up period, suggesting short-term preservation of masticatory function. A statistically significant effect of time was observed for tongue pressure, with moderate magnitude; however, this effect showed considerable inter-individual variability and limited consistency across participants. Within the limitations of this study, these findings indicate a low potential for clinically detectable functional impact under the evaluated conditions. Nevertheless, the results are restricted to short-term observation and should not be extrapolated to long-term functional outcomes or safety. Further controlled studies with larger samples and longer follow-up periods are required.

## Figures and Tables

**Table 1:** Mean values (±standard error), 95% confidence intervals (CI), and effect sizes (η^2^p) of maximum molar bite force (N) at baseline (I), 15 days (II), 30 days (III), and 60 days (IV) after hyaluronic acid injection in the mandibular angle.

Force	Periods								F	*p *	η^2^p
	I	95% CI	II	95% CI	III	95% CI	IV	95% CI			
Right	299.46 ± 45.79	209.87–389.03	321.13 ± 37.27	247.91–394.17	277.41 ± 47.27	182.54–370.14	256.56 ± 36.67	184.64–328.50	0.44	0.61	0.06
Left	300.24 ± 36.09	229.61–371.13	294.55 ± 27.65	240.47–349.01	341.30 ± 57.38	228.71–454.07	299.65 ± 54.23	193.30–406.12	0.50	0.68	0.05

Statistical analysis was performed using repeated-measures ANOVA with Bonferroni correction. Effect sizes are reported as partial eta squared (η^2^p). *p*-value (The significance level was set at *p* < 0.05). 95% CIs are presented to indicate the precision of the estimates. F = F-statistic (analysis of variance).

**Table 2:** Mean values (± standard error), 95% confidence intervals (CI), and effect sizes (η^2^p) of orofacial tissue pressure (kPa) at baseline (I), 15 days (II), 30 days (III), and 60 days (IV) after hyaluronic acid injection in the mandibular angle.

Pressure	Periods								F	*p*	η^2^p
	I	95% CI	II	95% CI	III	95% CI	IV	95% CI			
Lips	27.27 ± 5.36	15.42–39.12	31.83 ± 5.54	20.31–43.35	30.27 ± 4.52	20.79–39.75	37.38 ± 5.13	26.63–48.13	0.82	0.49	0.08
Tongue	43.21 ± 5.62	30.49–55.94	36.70 ± 5.34	24.61–48.80	34.12 ± 6.05	20.43–47.83	48.55 ± 6.56	33.70–63.41	5.90	0.03	0.39
Right cheek	16.67 ± 3.48	10.50–22.84	18.05 ± 3.89	11.70–24.40	16.90 ± 3.38	10.80–23.00	18.75 ± 3.02	12.80–24.70	0.23	0.87	0.02
Left cheek	21.80 ± 3.04	16.20–27.40	21.49 ± 4.89	13.80–29.18	20.18 ± 3.36	14.30–26.06	21.75 ± 2.31	16.90–26.60	0.08	0.96	0.01

Statistical analysis was performed using repeated-measures ANOVA with Bonferroni correction. Effect sizes are reported as partial eta squared (η^2^p). The significance level was set at *p* < 0.05. 95% CIs are presented to indicate the precision of the estimates. F = F-statistic (analysis of variance).

## Data Availability

The original contributions presented in this study are included in the article. Further inquiries can be directed to the corresponding author.
